# Socio-demographic and facility-based determinants of perceived quality of nutrition Services of Pregnant and Lactating Adolescent Girls in Trans-Mara east Sub-County, Narok County, Kenya

**DOI:** 10.1186/s40795-019-0316-5

**Published:** 2019-11-06

**Authors:** David Omondi Okeyo, Sussy Gumo, Elly O. Munde, Charles O. Opiyo, Zablon O. Omungo, Maureen Olyaro, Rachel K. Ndirangu, Nanlop Ogbureke, Sophie Efange, Collins Ouma

**Affiliations:** 1 Kenya Nutritionists and Dieticians Institute, Nairobi, P. O. Box 20436-00100 Kenya; 2 Department of Religion, Theology and Philosophy, Maseno University, School of Arts and Social Science, Maseno, Kenya; 3 Christian Aid-UK, P. O. Box 13864-00800, Nairobi, Kenya; 4 Christian Aid-UK, 35 Lower Marsh, London, SE1 7RL UK; 5 Department of Biomedical Science and Technology, Maseno University, School of Public Health and Community Development, Maseno, Kenya

**Keywords:** Quality, Nutrition, Facility-based determinates, Lactating, Pregnant, Adolescent

## Abstract

**Background:**

It has been established that use and utilization of nutrition services among adolescents are highly linked to availability, access, cost and quality of care. The main objective of this study was to assess the socio-demographic and facility-based factors as proxies to access to perceived quality of nutrition-specific and nutrition-sensitive services among adolescents in Trans-Mara East Sub-County, Narok County.

**Methods:**

The study adopted a cross-sectional approach that employed mixed methods on 291 households. Probability proportionate to size sampling techniques using cluster and simple random methods were used to practically access adolescents who are pregnant or lactating. Data were collected using questionnaires, in-depth interview and Focus Group Discussion. Quantitative data was analyzed descriptively using frequencies and inferentially using odds ratio and Z-test. Framework analysis was employed to analyze qualitative data.

**Results:**

A nutritionist was more likely to increase overall utilization (considered as a proxy index to access quality nutrition-sensitive and -specific services) by 3.18 times (OR = 3.18, 95% CI = 1.50–6.60, *P* = 0.002) and nurses 2.7 times (OR = 2.70, 95% CI = 1.40–5.30, *P* = 0.005). Generally, 80.7 and 69.4% attached positive value to environmental and basic personal hygiene, respectively, as being areas of nutrition-sensitive service delivery with a significant number higher than expected frequency of 50% (*P* < 0.05). An assessment of facility networks isolated only public health center as the key determinant of overall utilization. Public health centers among other health facilities were more likely to increase utilization (OR = 4.52, 95% CI = 1.50–13.50, *P* = 0.007). Assessment of distance to facility identified both distances as key determinants of overall utilization as those resident < 1 km from the facilities were 2.4 times more likely to utilize the facilities (OR = 2.42, 95% CI = 1.20–4.80, *P* = 0.012) while those resident 1-5 km were 5.3 times more likely to utilize the services (OR = 5.34, 95% CI = 1.90–15.10, *P* = 0.002) relative to longer distances. Finally, on methods of conveying messages, those who received messages through Information Education and Communication (IEC) materials were 7.8 times (OR = 7.85, 95% CI = 1.50–40.50, *P* = 0.014) and through face-to-face were 3.9 times more likely to utilize the services (OR = 3.91, 95% CI = 1.30–11.90, *P* = 0.016).

**Conclusion:**

Critical facility-based determinants of utilization of nutrition services include personnel (mainly nutritionist and nurse), distance and IEC materials.

## Introduction

Globally, there are limited data specific to access and utilization of nutritional advice and services of adolescents. Use and utilization of nutrition services among adolescents are highly linked to self-esteem, poverty, health beliefs, customs of the community in which they belong, the social structure and the level of education [1]. The level of education plays a significant role in the access to and utilization of nutrition services as confirmed by studies conducted in developing countries which attempted to isolate maternal education as one of the most important determinants of utilization of nutrition services, after controlling for other factors [1, 2]. The net effect of education on access and utilization of nutrition services among adolescents is significantly associated with young age at marriage and early childbearing. Education not only improves communication but also makes the adolescent to be confident enough to make decisions that affect their nutrition status [3]. Moreover, education imparts a feeling of self-worth and self-confidence, traits which are both critical in achieving changes in nutrition-related behavior [4, 5].

Socio-economic status has also been associated with significant effect on the utilization of maternal nutrition services [4, 5]. Adolescent from households that are wealthy are more likely to use nutrition services than those from poor households [4, 5]. Limited access and utilization of nutrition services among poor households could be due to the low priority given to health-seeking behaviour as compared to other daily basic needs, less formal education, unemployed, and detachment from social networks [6, 7].

Inadequate numbers of skilled health care workers and medical resources tend to mask the provision of appropriate quality of maternal healthcare [8]. This implies that quality of nutrition services offered by health personnel could be a proxy indicator to access and utilization of such services among adolescents, consequently leading to poor access and utilization of nutrition services among adolescents. However, this implication may be acceptable with caution since at times workforce and resources may be limited but of high quality.

In Narok County, 40% of girls aged 15–19 years were found to begin child-bearing in a Kenya Demographic Health Survey [9]. This was almost two times higher than the Kenyan national level (18%), yet to date, no study has assessed the socio-demographic and facility-based perceived quality of nutrition services in determining adolescents’ access and utilization of nutritional advice and services in Trans-Mara East Sub-County, Narok County. It is against this background that the aim of the current study focused on elucidating the socio-demographic and facility-based perceived quality of nutrition services in determining adolescents’ access and utilization of nutritional advice and services in Trans-Mara East Sub-County, Narok County, Kenya.

## Methods

### Study setting and research design

This study was conducted within Narok County where 40% of girls aged 15–19 years have begun child-bearing. In this region of study, 7.4% of adolescents are pregnant with their first child and 33% have ever given birth as compared to the national levels of 3.4 and 14.7%, respectively. These statistics are supported by the risks facing adolescents in Kenya which include but not limited to: high HIV infections, particularly among girls (16% of people living with HIV are aged 10–24 years); high teenage pregnancies (18%); early marriages (11%) for older adolescents (15–19 years); persistent female genital mutilation (11%); high rates of anaemia (41%) among pregnant adolescents; high number of adolescents exposed to sexual violence (11%) and physical violence (50%) as well as low secondary school attendance with a net ratio of 47%. All these risks perpetuate further the vulnerability of this age group to a healthy life.

The study was carried out in Trans Mara East Sub-County within Narok County. Trans Mara East Sub-County was purposively selected since it is the smallest in size (275.4 km^2^), among the four sub-counties in Narok County and had the highest prevalence of teenage pregnancies [9]. To achieve the objectives of this formative study, a cross-sectional study employing concurrent nested quantitative priority mixed methods approaches with both quantitative and qualitative research techniques was applied as previously reported in our work [10]. In this case, quantitative data were given priority while qualitative data were applied to triangulate some relevant quantitative components.

### Study population and sampling technique

#### Study population

The primary study population comprised of pregnant and lactating adolescent girls (aged 10–19 years old) resident in Trans Mara East Sub-County.

#### Sample size determination for quantitative approach

Sample size was determined using the Cochran formula [11], which allowed for calculation of an ideal sample size given a desired level of precision, desired confidence level, and the estimated proportion of the attribute present in the population. A total sample size of 292 was applied as previously reported in our work [10]. As previously indicated, proportionate distribution was done across 25 clusters equivalent to villages and by adolescent status (i.e. pregnant or lactating) to select study participants.

#### Test for sample size adequacy

Given the nature of the questionnaire where 90% of key variable measures were based on 5 point-Likert scale, descriptive test for sample size adequacy was performed using Kaiser-Mayor Olkin and Batt-test of sphericity as previously described [10].

#### Sampling procedure

Sampling was carried out as described in our work [10]. In brief, probability sampling techniques using cluster and simple random methods was used to practically access pregnant and lactating adolescents. An enumerator covered at least one village in a day to administer at least eight (8) questionnaires at random. Each enumerator moved to the center of a village selected for the day and began by facing North direction. After that, eight papers representing North (N), North East (NE), East (E), South East (E), South (S), South West and West (W) were shaken to give each direction equal chance of being selected and one piece finally picked to inform the direction to walk. Once direction was picked, an enumerator walked on a straight line to the next household until he/she reached a household with an eligible adolescent. Once the first adolescent was interviewed the enumerator again stood at the door of the just completed house facing North again and picked a direction from the pieces of papers randomized. The enumerator again walked to the next household. This process was repeated throughout the day until all eight adolescents were interviewed. An enumerator that reached the end of the village before completing the numbers required would go back to the centre of the village and randomly select a new direction to walk to. In case an enumerator double-selected the previous household, that household was passed until another eligible adolescent was reached. Each time an enumerator strived to interview three adolescent who were pregnant against seven who were lactating. This was to allow for proportionate distribution as per the target group listing ratio.

### Methods of data collection tools and process

Quantitative data was collected using adolescent questionnaire targeting critical indicators of access, utilization and individual power dynamics (See Supplementary File I). Focus Group Discussion guide was administered to adolescents and Community Health Volunteers (CHVs). For quality control purposes, the data enumerators were trained on the procedures and ethical issues related to the data collection and the instruments were pre-tested prior to use. The collection of data was performed under the supervision of the principal investigators. In each case, Kipsigis (local language), Kiswahili or in exceptional cases, English, was used as medium of communication.

#### Questionnaire-interview method

The questionnaire was administered to each respondent by an enumerator for a period of about 45 min. The focus was given to participants’ value label attached to critical items such as Collection and use of Iron and Folic Acid Supplements (IFAS), Regular nutrition assessment, Practice of quality of diet, Use of Ready to-Use Therapeutic Supplements/Ready to Use Supplementary Feeds (RUTS/RUSF), Vitamin A supplementation for the child, Use of Insecticide Treated Nets (ITNs), Regular visit for Nutrition education and counselling and overall adherence to utilization. These were treated generally as proxy quality indicators. Utilization pattern associated with nutrition services was assessed in such a way that participants who scored 4 or more items against a scoring rating between 4 and 5 were labelled ‘good utilizers’ while those who scored between 1 and 3 were labelled ‘bad utilizers. Good utilizers were assumed to have high chances of accessing quality of nutrition services. Validation of all proxy quality indicators were done based on principle component factoring and all the indicators revealed uni-dimentionality of measures.

#### Focus group discussions (FGDs)

Three focus groups targeting Community Health Workers, Parents (a group of biological parents of adolescents who either pregnant or lactating) and Mother-to-Mother Support Group (a group of adolescent pregnant or lactating) were conducted to understand issues surrounding nutrition needs of the adolescent girls who are pregnant or lactating. Focus was given to quality of the services received. Each target group was made up of an average of eight (8) members to engage in free discussions. Focus group discussions (FGDs) were conducted by a trained facilitator who also acted as moderator and a note-taker. All discussions were audio-recorded using digital recorder and transcribed verbatim. Major questions of FGDs included who provides nutrition advice and services for adolescent pregnant/lactating for adolescent pregnant/lactating at the health facility? A mention of some of the facilities’ nutrition pieces of advice/services provided for adolescent pregnant/lactating mothers; how accessible are these facilities and how are the nutrition advice information conveyed to the adolescent pregnant/lactating whenever they visit a facility to seek services? Finally, we asked them to gauge the level of satisfaction with the nutrition and health information provided to the adolescents.

### Statistical analyses

*Quantitative data analysis* adopted use of descriptive and inferential statistics. Descriptive statistics was used to characterize different frequencies. Z-test for single proportions was used to test for significant difference between the actual frequencies and expected frequency. Expected frequency was set at 50% for dichotomized data and 100/n percent for data that had more than two options. Principal Axis Factoring was used to establish the access pattern as well as generating *Batt*-scores for further modeling especially for indicators that were fitted into access and utilization models to determine cause and effect. Bivariate analysis based on odds ratio were done to determine how each of the socio-demographic and facility-based variables relate with perceived quality of nutrition services among pregnant and lactating adolescent girls. Expected frequencies for categorical data proportions were estimated as a ratio of 100% to the number of possible categories for each variable.

#### Diet diversity score

A simple dietary diversity score was used to predict micronutrient adequacy of diets of women of reproductive age. The food groups considered in the score for the Women Diet Diversity Score (WDDS) put more emphasis on micronutrient intake [10] than on economic access to food. A score based on nine food groups was chosen. Consumption recall was within 24 h food consumption period. The final score grouped the scores into highest category (> 6 food groups), medium (4 and 5 food groups) and Low (at most 3 food groups).

*Qualitative Data Analysis* on the other hand adopted the use of *Framework* analysis [12] for Focus Group Discussions. One key advantage with this framework analysis is that although it uses a thematic approach, it allows themes to develop both from the research questions and from the narratives of research participants. The process of data analysis began during the data collection, by skillfully facilitating the discussion and generating rich data from the interviews and FGDs, complementing them with the observational notes and typing the recorded information. This stage was followed by familiarization with the data, which was achieved by listening to voice records, reading the transcripts in their entirety several times and reading the observational notes taken during and after the interview and/or FGDs. The aim was to immerse in the details and get a sense of the interview as a whole before breaking it into parts. The next stage involved identifying a thematic framework, by writing *narrative memos* in the margin of the text in the form of short phrases, ideas or concepts arising from the texts and beginning to develop categories. At this stage, descriptive statements were formed and an analysis carried out on the data under the questioning route. The third stage, *indexing*, comprised sifting the data, highlighting and sorting out quotes and making comparisons both within and between cases. The fourth stage, *charting*, involved lifting the quotes from their original context and re-arranging them under the newly-developed appropriate thematic content.

## Results

### Response rate

The final sample size obtained was 337 adolescents who were either pregnant or lactating. This surpassed the minimum sample size required for the study.

### Socio-demographic characteristics of adolescent who are pregnant or lactating and quality-oriented overall utilization of nutrition services as proxy to access to quality nutrition services

The study assessed nutrition and health status of adolescents who were either lactating (69.1%) or pregnant (30.9%) in Trans Mara East Sub-County. Table 1 presents the socio-demographic characteristics of the study participants. The study established that 69.1% of adolescent were lactating while 30.9% percent were pregnant. Among these adolescents, 58.8% were single and living with parents/guardian, 37.4% were married, 2.1% were separated while only 1.8% were single and living alone. Majority of the adolescents had stopped going to school (30.3%), others had completed primary school (27.9%), were still attending primary school (10.7%) while others were still attending secondary school (22.0%). Collectively, it implied that 32.7% of the adolescents in this study were still attending school. Those who completed secondary (8.0%) and were still ongoing in tertiary (1.2%) made a total of 9.2%. The adolescents reported that 95.0% were Christians while only 2.7% were aligned to traditional religion. Further observation also revealed that adolescent’s food sources dominantly depended on crop growing (71.6%), purchase from market (55.8%), food aid (3.9%), donations from friends and neighbors (4.2%), donation from church (0.6%) and dependency from parents (50.1%). Further analysis revealed that all the socio-demographic indicators had no influence on the overall level of utilization except dependency from parents/guardian which was more likely to reduce the level of utilization by 0.6 times [Odds Ratio (OR) = 0.62, 95% CI = 0.39–0.99, *P* < 0.05].

**Table 1:** Socio-demographic characteristics of adolescent who are lactating and pregnant

Characteristics (*n* = 337)	Frequency	Percentage	OR	95% CI
Age [Mean (SD) = 16.3 (2.2)years]	337	–	0.91	0.77–1.09
Adolescent status				
Lactating	233	69.1	1.00	–
Pregnant	104	30.9	0.49*	0.29–0.81
Marital status				
Single living with Parent/Guardian	198	58.8	1.00	–
Married	126	37.4	1.12	0.19–6.50
Separated	7	2.1	0.74	0.16–3.60
Single living alone	6	1.8	0.97	0.58–1.66
Highest level of education				
Primary Completed	94	27.9	1.00	–
Primary Ongoing	36	10.7	1.83	0.18–18.40
Secondary Ongoing	74	22.0	2.88	0.29–28.60
Secondary Completed	27	8.0	6.00	0.56–64.00
Stopped Going to School	102	30.3	3.52	0.35–35.00
Tertiary Ongoing	4	1.18	–	–
Religion				
Others, Specify	14	4.2	1.00	–
Christian	323	95.8	0.44	0.04–5.30
Source of food				
Own agricultural production	240	71.6	1.00	–
Purchase from market	187	55.8	1.44	0.93–2.27
Food aid	13	3.9	0.75	0.23–2.49
Donation from neighbors and/or friends	14	4.2	1.11	0.36–3.42
Church donation	2	0.6	1.66	0.09–30.60
Dependency on parent or guardians	168	50.1	0.62*	0.39–0.99

Frequencies and percentages were determined as descriptive statistics for all categories of the socio-demographic characteristics. * *P* ≤ 0.05, logistic regression analysis; OR = Odds Ratio; Ref = reference group

### Perceived quality of nutrition services

Perceived quality of nutrition service delivery was assessed to demonstrate the extent to which services and nutrition needs were being met by service providers. Priority rating was given to perceived positive approval weighed by the magnitude of frequency based on the tallies of adolescents who agreed that such services were being satisfactorily offered. Generally, environmental hygiene (80.7%) and basic personal hygiene (69.4%) registered significant positive approval higher than expected frequency of 50% (*P* < 0.05). These domains of service were in the category of nutrition-sensitive issues.

Regular nutrition assessment both at ante-natal and post-natal (54.9%) and provision of IFAS (53.1%) were positively evaluated by slight majority. Adolescent services that performed significantly below the expected average prevalence included lactation management and processes (5.6%), access to nutrition supplements (17.5%), referrals for malnutrition (16.6%), nutrition support (26.1%), deworming (30.9%), regular follow ups (32.6%) and sexual and reproductive health sensitive to nutrition (48.7%) (Fig. I).

**Fig. 1:**
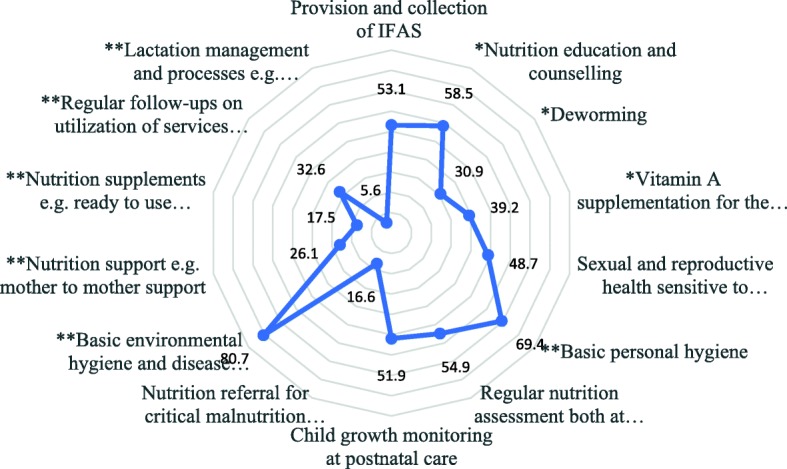
Distribution of adolescent who are lactating and pregnant by positive rating of service domains**.** The figure presents the different percentages of adolescents (pregnant and lactating based on positive rating of the service domains. * *P* < 0.05; ** *P* < 0.01; *** *P* < 0.001 based on z-test effect size

Further analysis to qualitatively explore the extent to which the adolescents were satisfied with nutrition services was mainly assessed by adolescents’ mothers in a focus group discussion (FGD). In the FGD as captured below, the general response demonstrated elaborated contentment with the service provision. However, the nature of contentment only displays psychological comfort and motivation which had nothing to do with nutrition-specific issues.

*R6- Yes in reality when I was pregnant, I was advised and given morale and due to that I was really very happy...*

*R2- Yes, I think they are satisfied because after talking with the doctor, she will realize it is normal to everyone and she can find support from community at large.*

### Quality of food based on diet diversity

Quality of food was based on previous diet diversity classification [13]. Adolescents who were pregnant or lactating isolated three forms of classification. High quality mark was assigned to > 6 food groups where only 1.5% of adolescents confirmed to have been met. Significant majority of adolescents (61.7%), expectant or lactating, registered medium mark after meeting 4–5 food groups (*P* < 0.05). Low mark (at most 3 food groups) was registered by 36.8% which was significantly below the expected frequency of 33.3% (Average percent for each category for the variable i.e. 100%/3).

### Facility-based determinants to overall utilization of critical services and products related to pregnant and lactating adolescents and their children

Overall adherence to utilization as proxy to quality care was assessed by dichotomizing the seven service domains (i.e. *Collection and use of IFAS, Regular nutrition assessment, Practice of quality of diet, Use of RUTS/RUSF, Vitamin A supplementation for the child, Use of ITNs, Regular visit for Nutrition education and counselling and overall adherence to utilization*) such that adolescents who scored 4 and 5 consistently on 4 items of adherence were weighted as 1(one) while those who scored the same on less than three items were weighted as 2 (two). Logistics regression model with overall adherence as dependent variable was established to determine how facility factors influence adherence based on odds ratios (Table 2). Results demonstrated that overall utilization of critical nutrition services depended on some selected indicators of service provider, facility type, distance and methods of conveying nutrition advice by service providers.

**Table 2:** Facility-related determinants of overall utilization of critical services and products

Factor	OR	95% CI
Service provider		
#Nutritionists	3.18*	1.50–6.60
Nurse	2.70*	1.40–5.30
Clinical Officer	1.64	0.70–3.70
CHVs	1.08	0.50–2.20
Community Development Social Worker	0.29	0.10–1.50
Pharmacists	0.39	0.06–2.70
Type of facility		
#Public dispensaries	1.27	0.60–3.00
Private clinic	0.44	0.20–1.30
Private hospital	1.84	0.10–27.10
Public hospital	0.25	0.01–6.50
Public Health Centre	4.52*	1.50–13.50
CBO and NGO health project	0.59	0.20–1.80
FBO project	1.61	0.40–6.10
At school	0.81	0.40–1.70
Distance facility		
# < 1 km	2.42*	1.20–4.80
1-5 km	5.34*	1.90–15.10
Methods of conveying messages		
#Video clips	1.00	–
IEC materials e.g. brochures leaflet etc.	7.85*	1.50–40.50
Face-to-face	3.91*	1.30–11.90
Social media e.g. WhatsApp and Facebook pages	0.29	0.10–1.40
Radio broadcasting	1.22	0.70–2.30

*P ≤ 0.05, logistic regression analysis; *CI*=Confidence Interval; *OR* = Odds Ratio. *CHVs* = Community Health Volunteers; *CBO*=Community-based organizations; *NGO*=Non-governmental organizations; *FBO*=Faith-based organizations; *IEC* = Information Education and Communication. For the initial regression analyses, the factor marked # was used as the reference group

Within service provider framework, both a nutritionist and nurse emerged as critical determinants of overall utilization of services. A nutritionist was more likely to increase overall utilization by 3.18 times (OR = 3.18, 95% CI = 1.50–6.60, *P* = 0.002). Likewise, a nurse was more likely to increase overall utilization by 2.7 times (OR = 2.70, 95% CI = 1.40–5.30, *P* = 0.005). Other cadres had no significant influence on utilization.

An assessment of facility networks isolated only public health center as the key determinant of overall utilization. Public health centers were more likely to increase utilization by 4.52 times (OR = 4.52, 95% CI = 1.50–13.50, *P* = 0.007). The other types of health facilities other than public health centres had no influence on overall utilization of adolescent’s nutrition services (Table 2). Further assessment of the distance to facility identified both distances as key determinants of overall utilization as those resident < 1 km from the facilities were 2.4 times more likely to utilize the facilities (OR = 2.42, 95% CI = 1.20–4.80, *P* = 0.012) while those resident 1-5 km were 5.3 times more likely to utilize the services (OR = 5.34, 95% CI = 1.90–15.10, *P* = 0.002). Finally, on methods of conveying messages, those who received the messages through IEC materials were 7.8 more likely to utilize the services (OR = 7.85, 95% CI = 1.50–40.50, *P* = 0.014) while for those who received the messages through face-to-face, they were 3.9 times more likely to utilize the services (OR = 3.91, 95% CI = 1.30–11.90, *P* = 0.016) (Table 2).

## Discussion

### Socio-demographic factors and quality of nutrition services

The current study was designed to elucidate the socio-demographic and facility-based perceived quality of nutrition services in determining adolescents’ access and utilization of nutritional advice and services in Trans-Mara East Sub-County, Narok County, Kenya. The study revealed that most socio-demographic factors did not have any influence on the level of utilization of nutrition services. The most significant factors were more related to the status of the adolescent, for example, the lactating group were more likely not to utilize services as compared to pregnant ones. Furthermore, the dependency on parent/guardian somehow reduced the level of utilization. This outcome observably diverged from previous studies finding which isolated education level as a critical determinant of nutrition services [1–3]. The findings of the current study contradicted the practical norm on the issue of education [4, 5, 14] that provides evidence on positive health benefits associated with the level of education in the context of a socio-ecological model of health. This was unexpected since it is known that school education increases the mother’s knowledge in regards to biological facets of human beings, common health problems, and healthy habits as included in standard school curricula. Similarly, educated mothers are more likely to be able to read comprehensibly and thereby understand better. As such, they are expected to understand health education messages presented in mass media and through other methods more than the less-educated ones. This deviation from the norm in this study population could be more related to the fact that this population totally dissociate literacy levels from their health issues. Other subject characteristics listed in the results were insignificant. In this study group, assuming that the education level was comparable, other factors outside the socio-demographics may apparently play more role in determining access and utilization of the essential nutritional services.

### Quality of nutrition advice and services

Rating of quality of services provided to adolescents who are pregnant and lactating revealed general satisfaction from the adolescents themselves. However, adolescents individually demonstrated low positive rating for the number of common services sought at facilities thus demonstrating a knowledge gap among the availability of such services. Conversely, the services could be entirely missing within the community structures.

Assessment of actual quality based on diet diversity revealed a reasonable proportion of those that do not meet the minimum food groups (36.8%) within a recall period of 1 week. Diet diversity is a central factor in health of adolescent who are pregnant and lactating as it is a key contributor to good health nutrition status. Optimal growth and development of adolescence is a composite of a balanced diet with essential vitamins and minerals from a variety of foods. If pregnancy comes into play at this stage and the nutritional needs of the adolescents are not fully met, consequences such as nutritional deficiencies, birth defects and propagation of the undernutrition cycle to future generation becomes inevitable [15]. Alam (2010) defines dietary diversity as an intake of adequate foods from a variety of food groups for optimal health, whereas, at an individual level, dietary diversity reveals the dietary quality exhibited by the adequacy of micronutrients in a diet. At the household level, it reflects the measure of access to foods, that is, access to quality foods especially of animal origin for a number of days and/or week(s) [16]. This implies that the about a third of adolescents who did not seem to access adequate or quality diet were at high risk of malnutrition and may potentially not achieve optimal health.

Several studies from different developing countries showed that poor people often face difficulties in accessing a diversified diet. Access is key to utilization but awareness of these food-based dietary guidelines is critical in equipping adolescents with knowledge and a more promising effect than access [15, 17, 18]. In a previous study, lack of knowledge was positively correlated with poor eating practices of the adolescents and entry into pregnancy with iron deficiency anemia with concomitant episodes of micronutrient deficiencies that undesirably affects the health of the developing fetus [19]. On the contrary, their eating and lifestyle behaviors were inclined to factors such as personal and cultural beliefs, perceptions, peer influences, food preferences, media, cost of food and the way the child was brought up [19]. Adolescent nutrition programs should then emphasize on promoting access of health services through delivering knowledge on nutrition and empowering them on food and nutrition security at an individual and household level [18, 20]. This may be done by allowing adolescents to engage on small-scale entrepreneurial income generating activities. Youth oriented farm support would be very useful in this context. Further discussions with adolescents revealed attempts to improve their knowledge on quality food and healthy diet during pregnancy and lactation. This area of focus somehow endeavors to sanction the scientific arguments on the role knowledge has on access to quality nutrition services and hence good status among the adolescents.

### Facility-based determinants to quality-oriented overall utilization of critical nutrition services and products related to pregnant and lactating adolescents and their children

This study identified key critical facility-based determinants of nutrition service delivery. Two key service providers that were isolated as critical in nutrition included nutritionists and the nurse. This shows that the providers are the most effective change agents where more resources could be invested. Since a nutritionist is properly trained on technical issues, they would be most appropriate in-service delivery to the adolescents than any other cadres. A more direct implications of our findings is that investing more on nutritionists at health facility level would impact positively on overall utilization of services by the adolescents. This outcome seems to be in line with the argument which identified inadequate numbers of skilled health care workers as functions of appropriate quality of maternal healthcare [8].

Despite the fact that nurses would also impact positively on overall utilization of nutrition services, in many cases, they are only useful on matters of nutrition due to lack of enough nutritionists to be engaged to offer such services. As a matter of fact, a nurse is not adequately trained on technical matters of nutrition and would require enhanced capacity on this subject for them to act as a stop-gap measure on service deliveries.

Public health centres also appeared to positively influence utilization of nutrition services. This outcome may be explained by the fact that nutrition cadres are at the moment placed to operate at health centres level. This cadre is not yet deployed at dispensary level even though dispensaries are widely used. Somehow, distance of < 5 km, had potential to improve services that were utilized. This points out to the fact that resources associated with nutrition services need to be placed closer to the adolescents to improve access including deployment of personnel. Distance from the health facility has been associated with access and utilization of nutrition services among the adolescents in previous studies [4, 21, 22]. However, satellite facilities with appropriate personnel would bring more impact. In many community setups, dispensaries are close to users and as such, these facilities may only need upscaling of nutrition services to improve utilization. Reducing the distance to the nearest health care facilities, irrespective of the type of the provider, is likely to raise demand for health care services. However, this must be accompanied by the availability of necessary nutritional services and well-trained personnel.

Our study also showed that on methods of conveying messages, those who received the messages through IEC materials were 7.8 more likely to utilize the services (OR = 7.85, 95% CI = 1.50–40.50, *P* = 0.014) while for those who received the messages through face-to-face, they were 3.9 times more likely to utilize the services (OR = 3.91, 95% CI = 1.30–11.90, *P* = 0.016). It became apparent in the current study that IEC materials and face-to-face were effective in communicating information that enhanced utilization of nutrition services. IEC in the current set-up comprised of a range of approaches, activities, and outputs which were collectively used to convey nutrition-related messages. Within the health centres/clinics within our study site, there were posters hanging on the clinic walls, with a range of targeted interpersonal communication to the pregnant and lactating adolescents. This seemed to auger well with the face-to-face discussions as the approaches were well integrated to an enhanced education on existing nutrition programmes thus increasing utilization of the services. Kreps (1988) [23] posits that health information are integral in health decision taking, health care management and play a vital role in health-seeking behaviour. He points out that the information provided regularly through access of information have the potential to influence the audience as currently observed in the current study.

## Conclusion

The study revealed that coverage was below average and a positive weight attached to a wide range of nutrition-specific and -sensitive services for adolescents, which were being met. Nutrition-specific services that had below 50% coverage for positive weight included nutrition support characterized by mother-to-mother support group, vitamin A supplementation, food supplementation, lactation management and processes and nutrition referrals for critical malnutrition episodes. Nutrition-sensitive service area that received below average coverage for positive weight included deworming and regular follow-ups on utilization of services. Further examination of the practice of quality diet as a proxy of access to nutrition advise and services revealed that a higher proportion than expected frequency still do not meet the minimum number of food groups (at least 4 groups) to reach the medium diet diversity. Critical facility-based determinants of utilization of nutrition services include personnel (mainly nutritionist and nurse), distance and IEC materials. Information Education Communication need to be integrated during service delivery. Empowering adolescents who are pregnant and lactating on food and nutrition security at an individual and household level may be done by allowing adolescents to engage on small-scale entrepreneurial income generating activities. Other important issues to consider during interventions may include all categories of maternal health literacy for example functional-related (reading, writing, numeracy and basic understanding of health conditions), interactive-related (how women communicate with health care providers and others and apply this information to their health and the health of their child/ren) and critical components (analysing information and using it to exert greater control over their health and the health of their children at the individual and community levels) as proposed by Nutbeam [24].

## Supplementary information

**Additional file 1.** This is the questionnaire used to generate the quantitative data on this article which is provided as Additional file 1: S1

**Additional file 2.** These are the dataset supporting the conclusions of this article which is provided as Additional file 2: S2.
