# Supplementary material for: Socio-demographic and facility-based determinants of perceived quality of nutrition Services of Pregnant and Lactating Adolescent Girls in Trans-Mara east Sub-County, Narok County, Kenya
Source: BMC Nutr. 2019 Nov 6;5:48. doi: 10.1186/s40795-019-0316-5 (PMC7050766; doi:10.1186/s40795-019-0316-5)
Supplement: Supplementary file 1 — Additional file 1. This is the questionnaire used to generate the quantitative data on this article which is provided as Additional file 1: S1 [file 40795_2019_316_MOESM1_ESM.docx]

## **Supplementary File I: Adolescent Questionnaire**

|  | **FORMATIVE RESEARCH FOR THE PROJECT**  ‘**IMPROVING ADOLESCENT NUTRITION PROJECT IN NAROK COUNTY, KENYA’** | |
| --- | --- | --- |
|  | **ADOLESCENT QUESTIONNIAIRE** |  |
| Q | **Questions** | **Options** |
| 1.0 | IDENTIFICATION |  |
| 1.1 | Name of the Data Collector |  |
| 1.2 | Survey date (dd/mm/yy) |  |
| 1.3 | County |  |
| 1.4 | Sub-County |  |
| 1.5 | Ward | Olomasani |
|  |  | Mogondo |
|  |  | Kapsasian |
|  |  | Likerin |
| 1.6 | Cell phone number of respondents |  |
| 1.7 | Gender of household head | 1. Male |
|  |  | 1. Female |
| 2.0 | **RESPONDENT’S SOCIO- ECONOMIC AND DEMOGRAPHICS STATUS** | |
| 2.1 | Respondent’s Study ID |  |
| 2.2 | Adolescent status | 1. Pregnant |
|  |  | 2. Lactating |
| 2.3 | Marital status of the adolescent | 1. Married |
|  |  | 2. Single living a lone |
|  |  | 3. Widowed |
|  |  | 4. Separated |
|  |  | 5. Divorced. |
|  |  | 1. Single living with parent/guardian |
| 2.3 | The adolescent’s spouse Study ID | _____________________________ |
| 2.4 | The adolescent’s parent/guardian Study ID | _____________________________ |
| 2.5 | Age of the adolescent | _____________________________ |
| 2.6 | Total number of people living in the household where adolescent reside | _____________________________ |
| 2.7 | Last level of education attained | 1. University/tertiary on going |
|  |  | 1. Secondary completed |
|  |  | 1. Secondary ongoing |
|  |  | 1. Primary completed |
|  |  | 1. Primary on going |
|  |  | 1. Stopped going to school |
| 2.8 | What is your religion currently? | 1. Christian |
|  |  | 1. Muslim |
|  |  | 1. Traditional |
|  |  | 1. Hindu |
|  |  | 1. Other Specify ___________________ |
| 2.9 | What are your sources of food? …………………………… |  |
|  |  |  |
|  | 1. Own agricultural production ……………………………. | 1. Yes |
|  |  | 2. No |
|  | 2. Purchase from market …………………………………… | 1. Yes |
|  |  | 2. No |
|  | 3. Food aid…………………………………………………… | 1. Yes |
|  |  | 2. No |
|  | 4. Donation from neighbors and/or friends…………………... | 1. Yes |
|  |  | 2. No |
|  | 5. Church donation …………………………………………… | 1. Yes |
|  |  | 2. No |
|  | 6. Dependent on parent or guardians …………………... | 1. Yes |
|  |  | 2. No |
| 2.10 | What is your **main** current source of income? |  |
|  |  | 1.        Agriculture (crop growing) |
|  |  | 2.        Livestock herding |
|  |  | 3.        Casual Labor |
|  |  | 4.        Self-employed |
|  |  | 5.        Skilled labor |
|  |  | 7.        Salaried employment |
|  |  | 8.        Petty trade |
|  |  | 10.     Charcoal burning |
| 2.11 | What is your **average monthly** income? . | ____________________Ksh |
| 3.0 | **INDICATORS OF ACCESS TO NUTRITIONAL ADVICE AND SERVICES**  (Information booklet to be provided to enumerators during training) | |
| 3.1 | Have you received any nutrition advice in the past three months? | 1. Yes |
|  |  | 2. No (If no skip 3.3) |
| 3.2 | Have you received any nutrition service in the past three months? | 1. Yes |
|  |  | 2. No |
| 3.3 | What domain of nutrition advice did you receive in the past three months? (if yes question 3.1) |  |
|  | 1. Advice on Healthy diet/diet diversity | 1. Yes |
|  |  | 2. No |
|  | 1. Advice on exclusive breastfeeding | 1. Yes |
|  |  | 2. No |
|  | 1. Advice on nutrient supplementation | 1. Yes |
|  |  | 2. No |
|  | 1. Advice on food fortification and blending | 1. Yes |
|  |  | 2. No |
|  | 1. Advice on appropriate complementary feeding (for lactating mothers only) | 1. Yes |
|  |  | 2. No |
|  | 6. Any other _____________________________________ | 1. Yes |
|  |  | 2. No |
|  |  | 1. Yes |
|  |  | 2. No |
| 3.4 | What domain of nutrition service did you receive in the past three months? (if yes question 3.2) |  |
|  | 1. Provision and collection of IFAS | 1. Yes |
|  |  | 2. No |
|  | 1. Nutrition education and counseling | 1. Yes |
|  |  | 2. No |
|  | 1. Deworming | 1. Yes |
|  |  | 2. No |
|  | 1. Vitamin A supplementation for the child | 1. Yes |
|  |  | 2. No |
|  | 1. Sexual and reproductive health sensitive to nutrition e.g. family planning | 1. Yes |
|  |  | 2. No |
|  | 1. Basic environmental hygiene, and disease prevention e.g. provision of ITNs | 1. Yes |
|  |  | 2. No |
|  | 1. Basic personal hygiene | 1. Yes |
|  |  | 2. No |
|  | 1. Regular nutrition assessment both at antenatal and postnatal | 1. Yes |
|  |  | 2. No |
|  | 1. Child growth monitoring at postnatal care | 1. Yes |
|  |  | 2. No |
|  | 1. Nutrition referral for critical malnutrition episodes | 1. Yes |
|  |  | 2. No |
|  | 1. Nutrition support e.g. mother to mother support | 1. Yes |
|  |  | 2. No |
|  | 1. Nutrition supplements e.g. ready to use therapeutic/Supplementary foods RUTS/RUSF | 1. Yes |
|  |  | 2. No |
|  | 1. Regular follow-ups on utilization of services e.g. through community strategy programmes | 1. Yes |
|  |  | 2. No |
|  | 1. Lactation management and processes e.g. normally done using lactation charts pathways | 1. Yes |
|  |  | 2. No |
| 3.5 | Who provided pieces of advice or services mentioned 3.3 and 3.4? |  |
|  | 1. Nutritionists | 1. Yes |
|  |  | 2. No |
|  | 1. Nurse | 1. Yes |
|  |  | 2. No |
|  | 1. Physician/doctor | 1. Yes |
|  |  | 2. No |
|  | 1. CHVs | 1. Yes |
|  |  | 2. No |
|  | 1. Community Development Social Worker | 1. Yes |
|  |  | 2. No |
|  | 1. Pharmacists | 1. Yes |
|  |  | 2. No |
|  | 1. Other (Specify)___________________________________ | 1. Yes |
|  |  | 2. No |
|  |  | 1. Yes |
|  |  | 2. No |
| 3.5 | Where were the pieces of advice/services provided? |  |
|  | 1. Public dispensaries | 1. Yes |
|  |  | 2. No |
|  | 1. Private clinic | 1. Yes |
|  |  | 2. No |
|  | 1. Private hospital | 1. Yes |
|  |  | 2. No |
|  | 1. Public hospital | 1. Yes |
|  |  | 2. No |
|  | 1. Public Health Centre | 1. Yes |
|  |  | 2. No |
|  | 1. CBO and NGO health project | 1. Yes |
|  |  | 2. No |
|  | 1. FBO project | 1. Yes |
|  |  | 2. No |
|  | 1. Public health clinics | 1. Yes |
|  |  | 2. No |
|  | 1. At School | 1. Yes |
|  |  | 2. No |
| 3.6 | How far in the source of advice/service from your residence? |  |
|  | 1. Less than 1 km | 1. Yes |
|  |  | 2. No |
|  | 1. 1-3 km | 1. Yes |
|  |  | 2. No |
|  | 1. Above 3 but less than 5km | 1. Yes |
|  |  | 2. No |
|  | 1. 5-10km | 1. Yes |
|  |  | 2. No |
|  | 1. Above 10km | 1. Yes |
|  |  | 2. No |
| 3.7 | How were the nutrition advise information conveyed to you in the past three months? |  |
|  | 1. IEC materials e.g. brochures, leaflets etc. | 1. Yes |
|  |  | 2. No |
|  | 1. Bulk SMS | 1. Yes |
|  |  | 2. No |
|  | 1. Internet links referrals | 1. Yes |
|  |  | 2. No |
|  | 1. Face to face | 1. Yes |
|  |  | 2. No |
|  | 1. Video clips | 1. Yes |
|  |  | 2. No |
|  | 1. Social media e.g. WhatsApp and Facebook pages | 1. Yes |
|  |  | 2. No |
| 3.8 | Rate your level of adherence to utilization of the following critical nutrition and health services | *Choose the most appropriate choice by circling the correct option.*  1=Strongly disagree, 2=Disagree, 3=neither disagree/agree, 4=agree, 5=Strongly agree |
|  | 1. Collection and use of IFAS | 1 2 3 4 5 |
|  | 1. Regular nutrition assessment | 1 2 3 4 5 |
|  | 1. Practice of quality of diet | 1 2 3 4 5 |
|  | 1. Use of RUTS/RUSF | 1 2 3 4 5 |
|  | 1. Vitamin A supplementation for the child | 1 2 3 4 5 |
|  | 1. Use of ITNs | 1 2 3 4 5 |
|  | 1. Regular visit for Nutrition education and counselling. | 1 2 3 4 5 |
|  | 1. Overall adherence to utilization | 1. 4 or more items scoring 4-5t rating |
|  |  | 1. Less than 4 items scoring 1-3 rating |
| 4.0 | **ACCESS TO NUTRITIONAL ADVICE AND SERVICES RATING**  (Information booklet to be provided to enumerators during training)  I will read some statements here which may or may not affect you. Please give rating on your level of access to the following advice or services. | MAPPING SECTION  *Choose the most appropriate choice by circling the correct option.*  1=Strongly disagree, 2=Disagree, 3=neither disagree/agree, 4=agree, 5=Strongly agree |
| 4.1 | I have been provided very good quality services on: |  |
|  | 1. Provision and collection of IFAS | 1 2 3 4 5 |
|  | 1. Nutrition education and counseling | 1 2 3 4 5 |
|  | 1. Deworming | 1 2 3 4 5 |
|  | 1. Vitamin A supplementation for the child | 1 2 3 4 5 |
|  | 1. Sexual and reproductive health sensitive to nutrition e.g. family planning | 1 2 3 4 5 |
|  | 1. Basic environmental hygiene, and disease prevention e.g. provision of ITNs | 1 2 3 4 5 |
|  | 1. Basic personal hygiene | 1 2 3 4 5 |
|  | 1. Regular nutrition assessment both at antenatal and postnatal | 1 2 3 4 5 |
|  | 1. Child growth monitoring at postnatal care | 1 2 3 4 5 |
|  | 1. Nutrition referral for critical malnutrition episodes | 1 2 3 4 5 |
|  | 1. Nutrition support e.g. peer mother to mother support | 1 2 3 4 5 |
|  | 1. Nutrition supplements e.g. ready to use therapeutic/Supplementary foods RUTS/RUSF | 1 2 3 4 5 |
|  | 1. Regular follow-ups on utilization of services e.g. through community strategy programmes | 1 2 3 4 5 |
|  | 1. Lactation management and processes e.g. normally done using lactation charts pathways | 1 2 3 4 5 |
|  | **I have consistently received good nutrition information through/on:** |  |
|  | 1. Nutrition IEC materials e.g. brochures, leaflets etc. | 1 2 3 4 5 |
|  | 1. Nutrition information through Bulk SMS | 1 2 3 4 5 |
|  | 1. Nutrition information through Internet links referrals | 1 2 3 4 5 |
|  | 1. Nutrition information through Face to face | 1 2 3 4 5 |
|  | 1. Nutrition information through Video clips | 1 2 3 4 5 |
|  | 1. Nutrition information through Social media e.g. WhatsApp and Facebook pages | 1 2 3 4 5 |
|  | 1. Recommended food groups for expectant and lactating mother e.g. Variety depicted with at least ≤ 3 food groups (**low**), 4 and 5 food groups (**medium**), ≥ 6 food groups (**highest**) | 1 2 3 4 5 |
| 5.0 | **POWER DYNAMICS**  I will read some statements here which may or may not affect you. Please give the correct rating to reflect the true picture of your situation. | BARRIERS  *Choose the most appropriate choice by circling the correct option.*  1=Strongly disagree, 2=Disagree, 3=neither disagree/agree, 4=agree, 5=Strongly agree |
|  | **Self Esteem** |  |
|  | 1. I can resolve nutrition and dietary related problems on my own | 1 2 3 4 5 |
|  | 1. I depend on significant others to resolve nutrition and dietetics problems | 1 2 3 4 5 |
|  | 1. Whenever I want to seek nutrition services from a health facility and somebody opposes me I always push and get what I want | 1 2 3 4 5 |
|  | 1. I always get some way to deal with health and nutrition problems that confronts me. | 1 2 3 4 5 |
|  | 1. I can overcome my spouse/guardian/parents’ contrary decision to seek nutrition and health services | 1 2 3 4 5 |
|  | 1. I can take action to improve nutrition status through healthy eating during hard times | 1 2 3 4 5 |
|  | 1. My decision to seek health and nutrition services is always directed by significant others (e.g. parents, spouse, guardian, siblings) | 1 2 3 4 5 |
|  | 1. I always find it difficult to deal with health and nutrition problems that confronts me. | 1 2 3 4 5 |
|  | **Social Position** |  |
|  | 1. My spouse live with respects me and care about my nutritional health | 1 2 3 4 5 |
|  | 1. People I live with respects me and care about my nutritional health | 1 2 3 4 5 |
|  | 1. People in my community always care about my health and nutrition status | 1 2 3 4 5 |
|  | 1. People in my church always care about my health and nutrition status | 1 2 3 4 5 |
|  | 1. My father value my opinion | 1 2 3 4 5 |
|  | 1. My mother value my opinion | 1 2 3 4 5 |
|  | 1. My guardian/ value my opinion | 1 2 3 4 5 |
|  | 1. Members of extended families value my opinion | 1 2 3 4 5 |
|  | 1. My spouse/guardian/parents’ / members of extended families still show willingness to support my education despite pregnancy/lactation status | 1 2 3 4 5 |
|  | 1. My peers within the community demonstrate socio-support whenever I am in need. | 1 2 3 4 5 |
|  | 1. My guardian takes full responsibilities in educating me regardless of my status | 1 2 3 4 5 |
|  | 1. My father takes full responsibilities in educating me regardless of my status | 1 2 3 4 5 |
|  | 1. My mother takes full responsibilities in educating me regardless of my status | 1 2 3 4 5 |
|  | **Ability to decide on resource use** |  |
|  | 1. I can allocate money to seek health and nutrition services without permission from parents/spouse/guardian/siblings | 1 2 3 4 5 |
|  | 1. I am able to personally decide on which areas of my needs require resources to be allocated. | 1 2 3 4 5 |
|  | **Gender Dynamics** |  |
|  | 1. Members of this community have respect to pregnant/lactating adolescents | 1 2 3 4 5 |
|  | 1. Members of this community treat girls and boys equally | 1 2 3 4 5 |
|  | 1. Both pregnant/lactating adolescent girls and boys of same age group are given equal opportunity in youth leadership | 1 2 3 4 5 |
|  | 1. Both boys and pregnant/lactating adolescent girls of the same age group receive equal opportunity to pursue education | 1 2 3 4 5 |
|  | 1. Both boys and pregnant/lactating adolescent girls receive equal opportunity in resource allocation | 1 2 3 4 5 |
|  | 1. Cases of gender-based violence against girls who are pregnant or lactating are common in this community. | 1 2 3 4 5 |
|  | 1. Pregnant/lactating adolescent girls sometimes receive psychological, emotional and verbal abuse when they visit health facilities | 1 2 3 4 5 |
